# Probing the phenomics of noncoding RNA

**DOI:** 10.7554/eLife.01968

**Published:** 2013-12-31

**Authors:** John S Mattick

**Affiliations:** 1 **John S Mattick** is an *eLife* reviewing editor, and is at the Garvan Institute of Medical Research, Sydney, Australia and St Vincent’s Clinical School and the School of Biotechnology and Biomolecular Sciences, University of New South Wales, Sydney, Australiaj.mattick@garvan.org.au

**Keywords:** long noncoding RNAs, knockout mouse models, lethality, developmental defect, brain development, genetics, Mouse

## Abstract

Genetic knockout experiments on mice confirm that some long noncoding RNA molecules have developmental functions.

**Related research article** Sauvageau M, Goff LA, Lodato S, Bonev B, Groff AF, Gerhardinger C, Sanchez-Gomez DB, Hacisuleyman E, Li E, Spence M, Liapis SC, Mallard W, Morse M, Swerdel MR, D’Ecclessis MF, Moore JC, Lai V, Gong G, Yancopoulos GD, Frendewey D, Kellis M, Hart RP, Valenzuela DM, Arlotta P, Rinn JL. 2013. Multiple knockout mouse models reveal lincRNAs are required for life and brain development. *eLife* **2**:01749. doi: 10.7554/eLife.01749

**Image** A wild-type (left) and mutant (right) mouse seven days after birth; this particular mutation (Mdgt^−/−^) is partially lethal with growth defects in survivors

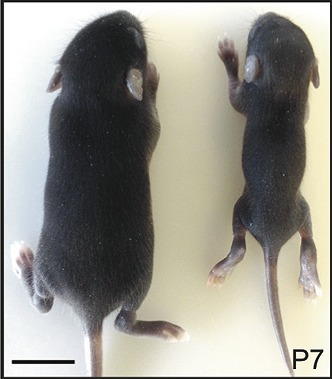

It has been known since the late 1970s that many DNA sequences are transcribed but not translated. Moreover, most protein-coding genes in mammals are fragmented, with only a small fraction of the primary RNA transcript being spliced together to form messenger RNA. For many years it was assumed that untranslated RNA molecules served no useful purpose but, starting in the mid-1990s, a small body of researchers, including the present author (Mattick, 1994), have been arguing that these RNAs transmit regulatory information, possibly associated with the emergence of multicellular organisms. This is supported by the observation that the proportion of noncoding genomic sequences broadly correlates with developmental complexity, reaching over 98% in mammals (Liu et al., 2013), although others have argued that the increase in genome size is due to the inefficiency of selection against non-functional elements as body size goes up and population size goes down (Lynch, 2007).

High-throughput sequencing analyses over the past decade have shown that the majority of mammalian genome is transcribed, often from both strands, and have revealed an extraordinarily complex landscape of overlapping and interlacing sense and antisense, alternatively spliced, protein-coding and non-protein-coding RNAs, the latter generally referred to as long noncoding RNAs (lncRNAs). Moreover, the repertoire of these lncRNAs is different in different cells (Carninci et al., 2005; Cheng et al., 2005; Birney et al., 2007; Mercer et al., 2012). While some transcripts may encode previously unrecognized small proteins, the function or otherwise of the vast majority of lncRNAs remains to be determined.

Because many lncRNAs appear to be expressed at low levels, and many have lower sequence conservation than messenger RNAs, one interpretation has been that these RNAs represent transcriptional noise from complex genomes cluttered with evolutionary debris. However, assessments of sequence conservation rely on assumptions about the non-functionality and representative distribution of reference sequences, which are not verified and cannot be directly tested (Pheasant and Mattick, 2007). Nonetheless, many lncRNAs show patches of relative sequence conservation (Derrien et al., 2012), and even more do so at the secondary structural level (Smith et al., 2013).

Expression analyses have shown that lncRNAs originate from all over the genome and are expressed at different times during differentiation and development (Dinger et al., 2008), often exhibiting highly cell-specific patterns (Mercer et al., 2008). The precision of lncRNA expression is consistent with evidence suggesting that many are associated with chromatin-modifying complexes, thereby acting as regulators of the epigenetic control of differentiation and development (Mercer and Mattick, 2013).

A number of lncRNAs have also been linked to complex diseases like cancer (Mattick, 2009) and other complex physiological processes (see, for example, Rapicavoli et al., 2013). However, these results seem at odds with the fact that few lncRNAs have been identified in traditional genetic screens. The reason for this is likely a combination of phenotypic, technical and expectational bias: mutations in protein-coding regions of the genome generally have phenotypes that are more severe, and are easier to identify, than those in non-coding regions. By contrast, in this context, it is worth noting that ∼95% of all variants associated with complex (as opposed to monogenic) diseases in humans map to non-coding, presumably regulatory, sequences (Freedman et al., 2011).

Still, the gold standard in this field is the targeted in vivo silencing or deletion of specific genes, and since few of these have been conducted to date, some researchers have remained sceptical about the biological significance of lncRNAs. Now, in *eLife*, John Rinn, Paolo Arlotta and co-workers at Harvard, MIT, the Broad Institute, Rutgers and Regeneron Pharmaceuticals—including Martin Sauvageau, Loyal Goff and Simona Lodata as joint first authors—report the results of the first large-scale attack on the question (Sauvageau et al., 2013). They selected 18 lncRNA genes in the mouse genome that had been stringently assessed for lack of protein-coding capacity and that did not overlap with known protein-coding genes or other known gene annotations—hence the name long intergenic noncoding RNAs (lincRNAs)—and generated knockout mouse mutants by replacing the lncRNA gene with a *lacZ* reporter cassette.

Sauvageau, Goff, Lodata et al. report discernable developmental problems in five of the 18 mutants, with three exhibiting embryonic or post-natal lethality, two of which exhibited growth defects in the survivors. The phenotypes of two of the mutants were analyzed in detail: one of the mutants that died showed defects in multiple organs (including the lung, heart and gastrointestinal tract), and one of the mutants that survived with growth defects also showed defects in the cerebral cortex. Other mutants that did not exhibit overt developmental defects showed brain-specific expression patterns and may be associated with cognitive defects that are not grossly apparent at the developmental level.

Another group (Grote et al., 2013) recently generated a different knockout allele for one of the 18 lincRNAs interrogated by Sauvageau et al., and also reported an embryonic lethal phenotype, albeit with some differences. Importantly, the approach used by Grote et al. also provided strong evidence that the mutant defects were not caused by an indirect effect on an overlapping genomic element, such as an enhancer for a nearby gene.

The work of Sauvageau, Goff, Lodata et al. is a mini tour-de-force that shows that there are lncRNAs with important developmental functions in vivo, and it joins a small number of studies from other pioneering groups that show the same thing (Lewejohann et al., 2004; Gutschner et al., 2013; Li et al., 2013), although not all of the targeted lncRNAs showed a phenotype. Similarly, other knockout experiments of widely expressed lncRNAs, as well as some of the most highly conserved elements in the mammalian genome, also did not yield discernable phenotypes (Ahituv et al., 2007; Nakagawa et al., 2011), which should sound a note of caution about the interpretation of negative results.

Indeed, since most lncRNAs are expressed in the brain (Mercer et al., 2008) and many are primate-specific (Derrien et al., 2012), it may be that much of the lncRNA-mediated genetic information in humans (and in mammals generally) is devoted to brain function, and therefore not easily detectable in developmental, as opposed to cognitive, screens. A good example is a noncoding RNA called BC1 that is widely expressed in the brain: knockout of BC1 causes no visible anatomical consequences, but it leads to a behavioural phenotype that would be lethal in the wild (Lewejohann et al., 2004).

Although evidence for the hypothesis that lncRNAs have a role in mammalian development, brain function and physiology is growing, there is also a clear need for more sophisticated and comprehensive phenotypic screens, especially with respect to cognitive function.
